# Plastic Optical Fibre Sensor for Spine Bending Monitoring with Power Fluctuation Compensation

**DOI:** 10.3390/s131114466

**Published:** 2013-10-25

**Authors:** Mohd Anwar Zawawi, Sinead O'Keeffe, Elfed Lewis

**Affiliations:** 1 Department of Electronic and Computer Engineering, University of Limerick, Plassey Rd., Limerick, Ireland; E-Mails: sinead.okeeffe@ul.ie (S.O.); elfed.lewis@ul.ie (E.L.); 2 Faculty of Electrical and Electronics Engineering, Universiti Malaysia Pahang, Pekan, Pahang 26600, Malaysia; E-Mail: mohdanwar@ump.edu.my

**Keywords:** plastic optical fibre sensor, spine monitoring, power fluctuation compensation, intensity modulation technique

## Abstract

This paper presents the implementation of power fluctuation compensation for an intensity-based optical fibre bending sensor aimed at monitoring human spine bending in a clinical environment. To compensate for the light intensity changes from the sensor light source, a reference signal was provided via the light reflection from an aluminum foil surface fixed at a certain distance from the source fibre end tips. From the results, it was found that the investigated sensor compensation technique was capable of achieving a 2° resolution for a bending angle working range between 0° and 20°. The study also suggested that the output voltage ratio has a 0.55% diversion due to input voltage variation between 2.9 V and 3.4 V and a 0.25% output drift for a 2 h measurement. With the achieved sensor properties, human spine monitoring in a clinical environment can potentially be implemented using this approach with power fluctuation compensation.

## 1. Introduction

The spinal column is an important element in the human physiological system. A human spine consists of three main regions, namely the cervical, thoracic and lumbar region. The cervical spine which connects the human skeleton with the rest of the human vertebral column is composed of seven cervical bones. Towards the lower part of the cervical spine are twelve thoracic bones which can rotate and undergo flexion movements. However, their connection with the rib cage prevents them from excessive flexion. The lowest part of the human spine is the lumbar area which consists of five bones and it is connected to the sacrum. The lumbar spine is more robust than the cervical and thoracic spines as its main function is to support the weight of the upper body area [1]. With its robustness and strength, it allows body movement in several directions, including flexion, extension, lateral rotation and lateral flexion (side bending).

There are several problems associated with poor spinal conditions such as abnormal kyphosis (over curvature of the thoracic spine), lordosis (excessive inward curvature of the lumbar spine) or scoliosis (side to side curve of the thoracic spine) [2]. These problems lead to the requirement of spine monitoring tools for physiotherapists and physicians to establish a standard measurement approach among people with back spine problems. In certain patient conditions, it is even becoming necessary to have a continuous measurement data of spine movement for several hours in order to provide sufficient information for them to have a thorough understanding of the problems [3].

In general, spine bending measurements can be implemented using several different devices. These devices can be categorized into three different types; hand-held type, non-contact type and skin-mounted type. The hand-held type devices are used manually by the examiner during the spine examinations. Usually there is no other device mounted on the skin-surface during the examination time. The process of measuring the spine posture is only possible while the subjects under testing are static. The recording of the spine angle at particular points is done either manually by the examiner or with the help of a portable transmitter. Some practical examples of this type of device are flexible rulers [4], tape measures [5], goniometers [6] and spinal mice [7].

Another different approach for spine monitoring are the so-called non-contact devices. The measurement process for this type of devices takes place at a distance from the body. The image of the human's back is captured using a camera. To improve the quality of detection images, these sensors would require the placement of reflective markers or a harness on the back area. A few examples of this non-contact type have been presented such as CCD video systems [8], rasterstereography [9] and digital video fluoroscopy [10].

For both device types described above, it is impractical to provide a continuous assessment of human spine bending movement considering the limitations of the approaches such as human examiner constraints to operate the device over a longer measurement duration as well as the risk and side effects associated with the assessment technique due to radiation exposure on the human skin [11]. To overcome these shortcomings, the implementation of skin-mounted devices for spine monitoring is important especially for long term assessment of the human spine condition of patients or any potential subject for an early diagnosis of back problems.

This paper investigates the development of a skin-mounted type device using a plastic optical fibre bending sensor based on the intensity modulation technique with power fluctuation compensation. The compensation was implemented by using the output voltage ratio between the sensor and reference fibre outputs. The effect of the light source intensity on the output ratio of the bending sensor was tested by monitoring the output ratio reading of the sensor due to several pre-determined input voltage levels. In addition, the output drift effect was also taken into consideration before the sensor properties such as resolution, working range and output linearity were determined.

## 2. Review of Skin Mounted Devices for Spine Monitoring

In general, skin-mounted devices for spine monitoring can be divided into four main groups according to their sensing mechanism approaches. Firstly, the spine bending movement is measured based on the elongation and contraction (strain and stress) between two points on the human back. For devices of this kind, the bending angle of the spine is determined indirectly via the strain measurement at particular points on the human skin surface. The main requirement of a strain sensor in this case is an efficient transfer of the applied load to the sensor elements. It is relatively more difficult to ensure an accurate bending measurement via strain analysis as the sensor is only attached on the surface of the human skin. To have an optimum stretch and compress effects on the strain sensor, it is the best to have the sensor embedded in the subject under study (human skin), which in this case no longer applicable for invasive spine bending monitoring. Another issue related to this strain-based sensor to measure spine bending is that strain can be so small that very high resolution sensors are required despite the apparently large deflection curvature of the spine [12]. The use of strain sensors for spine bending measurement has been presented utilizing strain gages on a thin stainless steel beam to measure lumbar flexion-extension with an accuracy of within 3° in both the sagittal and frontal planes [13].

Another alternative mechanism is applied by indirectly extracting the posture data from the acceleration of the spine during flexion and extension exercise movement. However, this method requires an additional sensor such as gravitometer to provide the data when the subject is in a static condition. In this spine bending measurement case, this type of sensor is more suitable to assess the velocity and acceleration of the spine during the body movement in flexion, extension and lateral directions or during body posture changes such as sitting, standing, walking and running. One good example of this type of sensor was presented using a piezoresistive accelerometer to record the acceleration data between trunk and thigh position during sit to stand movements [14].

The next type of skin-mounted device for spine monitoring application is based on the positional sensor approach. The sensor is normally made up of one base sensor and several other sensor pairs. The orientation measurement of other sensors with respect to the base sensor will provide a three dimensional coordinate frame of the human spine position. The sensor has to be reset prior to each measurement to store the default coordinate frame and to allow calculation of spine position for each movement. Several different positional sensors have been tested for spine monitoring application such as using an electromagnetic sensor—Isotrack^®^ [15] and a fibre optic sensor—Shapetape™ [16] with different degree of success. For example, the optical bending sensor using Shapetape™ is implemented based on Cartesian position and orientation vectors between each optical sensor pair and a base sensor to detect curvature and twist movements along the sensor length during static posture and dynamic movement of the patient [17].

Considering the limitation of the previous three types of devices to provide any means of compensation data for spine bending and movement in a single measurement during long period monitoring, a direct measurement of bending angle and spine movement using a curvature sensor is necessary. This can be realized using several approaches such as ultrasonic and optical fibre sensors.

In brief, an ultrasonic sensor for spine bending applications is composed of several pairs of transmitters and receivers. An ultrasonic movement analysis device called Zebris^®^ CMS 50 was used to record the angular displacement of the thorax and pelvis in humans during walking on a treadmill [18]. The sensor consists of an ultrasonic marker (transmitter) and three ultrasound microphones (receivers) attached at the back of the human body using adhesive tape. The level of the detected ultrasonic signal depends on the spine bending angle and direction between the marker and microphones.

The other type of curvature sensor for spine bending monitoring was tested using an optical fibre sensor integrated into a garment to monitor the spine while the subject was in a seated posture [19]. This sensor is composed of a light source, a light detector and a plastic optical fibre with an abraded area at one side of the fibre [20]. The sensor length is around 50–55 cm and is stitched to the outer surface of an elastic garment. The light intensity of the fibre is sensitive to the direction and degree of curvature of the bending at particular abraded areas along the fibre. The reproducibility of this kind of sensor is difficult to achieve if the side area of the fibre is polished manually as the light attenuation characteristics due to bending depend on the length and depth of the abraded side along the fibre [21]. Another issue is that no compensation method has been addressed in this sensor implementation which could lead to inaccurate bending angle measurement due to the power fluctuation.

## 3. Review of Related Compensation Approaches for Intensity Modulated Optical Fibre Sensors

For the intensity modulation technique in optical fibre sensors, the detection mechanism is basically based on the detection of optical intensity. As the output reading from the sensor might be affected by various noise sources coming from the light source, photodetector, external light coupling and other environmental factors, it becomes necessary to have a referenced or differential-balanced system to reject such fluctuations in the sensor measurement. Besides that, to minimize the effects of disturbance on the light intensity, the reference signal should be placed as close as possible to the sensor output.

**Figure 1.**
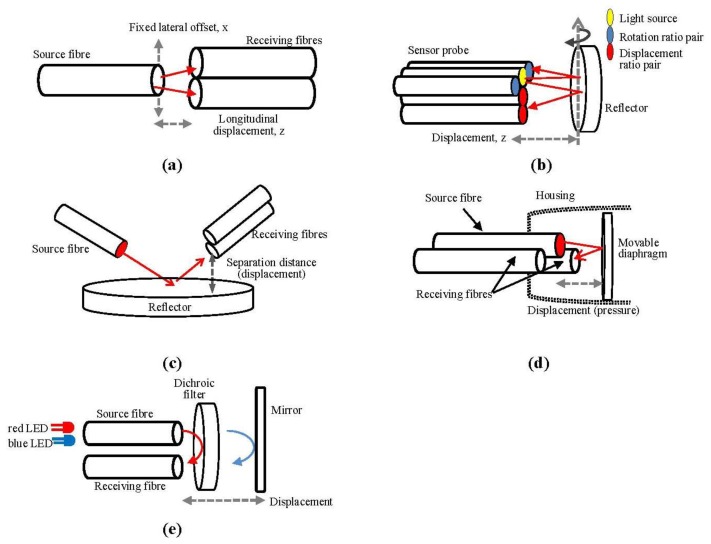
(**a**) Twin receiving fibres–transmission mode; (**b**) 2D-reflective sensing probe; (**c**) Twin receiving fibres–reflective mode; (**d**) Twin reflective fibres; (**e**) Dual wavelength inputs.

The basic approach to provide a compensation signal for light intensity fluctuation was presented using twin receiving fibre cables [22] as shown in Figure 1a. A white light lamp and a He-Ne laser source were separately used as the light sources. The output intensity ratio between these two receiving fibres was used to measure the displacement between the source fibre and twin receiving fibres.

As a comparison, Figure 1b shows another compensation technique which has been applied in 2D configuration to compensate for the light source intensity for measuring the displacement and rotation of a reflective object using a sensor probe [23]. The sensor consisted of one source fibre and another four receiving fibres placed in a ‘T’ figure. Two of the receiving fibres were used to sense the rotation of the reflector surface and the other two were applied to sense the changing distance between the reflector and the fibre tip surface by means of the signal ratio between these two fibres.

An alternative approach was presented by using a light source and two identical receiving fibres. The source fibre was placed in the opposite direction of the receiving fibres as in Figure 1c [24]. The light source was reflected by a mirror and entered the receiving fibres—fibres A and B—with different intensity due to the distance of the different fibre end tips to the mirror. The ratio of light intensity difference between the two fibres (A − B) and their summation (A + B) was used to compensate for the changes in light source intensity.

Another sensor compensation technique was described in pressure sensor application using one source fibre (middle) and two receiving fibres (front and behind) [25]. Each fibre end tip was placed at a different distance away from the reflecting target (diaphragm) as shown in Figure 1d. The ratio between the two outputs of these fibres was used to measure the position of the diaphragm and thus the pressure reading.

As shown in Figure 1e, the last compensation technique to be discussed here was presented using dual-wavelength inputs to measure the displacement between the reflector and the sensor surface. A blue LED (465 nm) and red LED (625 nm) were used in this configuration [26] and alternately switched on and off to supply the light source from the same source fibre. A dichroic filter with a cut-off wavelength of 500 nm was fixed 0.5 mm away from the fibre tip to reflect the red light into the receiving fibre and to pass the blue light from the source fibre to the mirror. The blue light intensity was changed as the mirror was moved away from the fibre end tips, while the red led which was constantly reflected by the dichroic filter became the reference output for the signal compensation of this sensor.

For all the compensation methods presented above, the implementations of the compensation signal were specifically for displacement [22–24,26], pressure [25] and tilt angle [23] measurements. The use of a mirror and dichroic filter to provide the reference signal for the compensation purpose seems to be more difficult to realize for the bending sensor configuration as the reflector or filter will also move due to the bending movement between the fibres which leads to an unreliable referencing signal. One possible option to realize a compensation technique is to use a 50:50 beam splitter element in between the input fibres and the sensor fibre. There are several beam splitter options available in the market such as the Pellicle beam splitter [27], Polka Dot beam splitter [28] and visible beam splitter [29]. However, due to the large diameter size of the splitter (minimum available size is 1 inch) it is not suitable for the intensity modulation application proposed in this investigation. The reason is that installing a large diameter of splitter into the fibre holder would require a bigger overall size of the sensor. This could lead to a stiff bending movement between the input fibres and the sensor fibre.

Another possible approach is to implement an optical coupler such as a face coupler, Y-coupler and polished coupler [30] along the source fibre before inserted it into the sensor for the bending measurements. For example, a 1:2 Y-coupler could be used to provide a reference signal at one of the coupler outputs while the other output could be adopted for the sensor measurement signal. Besides the significant loss from coupling effect, this technique is also incapable of compensating for any signal disturbance due to unwanted fibre bending and movement between the coupler output and source fibre end tip of the sensor. Thus, it is important to have a referencing feedback which is retrieved as close as possible to the sensor element. A different and relatively more suitable compensation approach is presented in this investigation considering the size of the sensor and the location of the reference fibre input for the implementation of bending sensor using plastic optical fibre to cancel out the effect of power fluctuation.

## 4. Sensor Configuration for this Investigation

The experimental setup of the sensor configuration for this investigation consists of a multimode plastic optical fibre LED (GHV-4001, Mitsubishi Rayon Co. Ltd., New York, NY, USA) with a core diameter of 1 mm, numerical aperture at 0.5 and step index profile, two bright green LEDs (IF-E93, Industrial Fiber Optics Inc., Arizona, AZ, USA) with a peak wavelength at 530 nm and spectral bandwidth of 50 nm and two photodiodes (SFH-250 V, Avago Technologies, San Jose, CA, USA & Singapore) with a photosensitivity spectral range between 400 nm and 1,100 nm. This sensor configuration was divided into two main fibre parts. The first part consists of three fibre optic cables, two of which were the input fibres connected to the green LEDs and the other one was the output fibre connected to the photodiode as a reference output. The other part was also another output fibre cable representing the sensor output for bending measurements.

**Figure 2.**
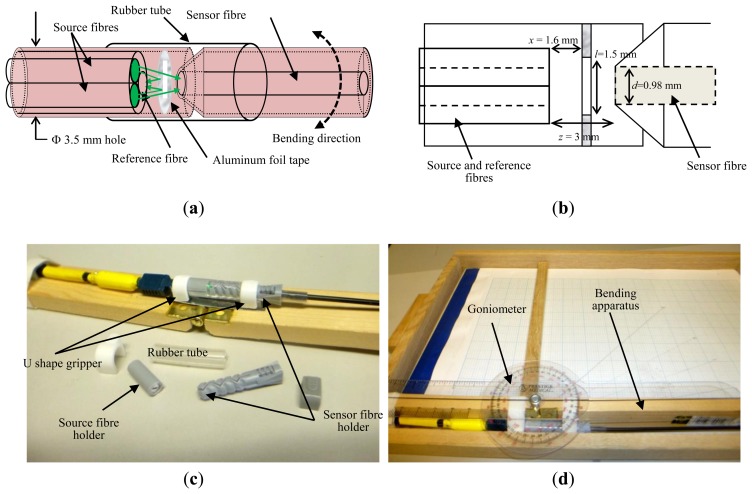
(**a**) Sensor configuration in this investigation; (**b**) Sensor cross section (side view); (**c**) Photo of the sensor placed on a wooden bend apparatus; (**d**) Bending test rig with side-attached goniometer.

As illustrated in Figure 2a, in the first part containing the source fibres and the reference fibre, all three of these fibres were merged together at the fibre end tips using a modified V-pin crimp connector. To firmly hold all the fibres at the remaining area of the fibres, these fibres were securely insulated using a shrink tube at a certain length. Then, the V-crimp connector containing all the three fibres was placed into a plastic tube with a center through-hole diameter of 3.5 mm. At the end of the opposite hole of the tube (near the end tips) a reflector was attached. A small window of 0.5 mm × 1.5 mm was cut out of the aluminum foil to allow certain portions of the light source to travel through the window into the sensor fibre of the sensor. The rest of the light source was reflected back into the reference output fibre via the reflective surface of the reflector. The other side of the reflector was a non-reflective surface so that the effect of back-reflection from the sensor fibre end tip surface into the reference fibre was minimized. A soft silicon rubber tube of 5 mm inner diameter was used to hold the first and second parts together during the bending movement.

During the measurement, the sensor was placed on the top of a self-made wooden bending apparatus as shown in Figure 2c to ensure a consistent sensor position at each bending angle and to allow angular movement in one direction only (thus lateral direction was avoided). Two U-shape grippers were used to hold the fibre at the both ends of fibre holders (source and sensor) without affecting the response of the fibre due to the tape fixing task. This bending apparatus was then placed on a test rig with a 2.5° bending scale between 0° and 20° to measure the sensor output voltages at several pre-determined bending angles. A goniometer was attached at the side area of the bending apparatus for bending angle reference during the measurement. Three different measurements were made in this investigation; power fluction assessment, bending assessment and repeatability assessment.

For fluctuation assessment the output voltage and its ratio with respect to the reference voltage were monitored at several different input voltages between 2.9 V and 3.4 V. The sensor was maintained in a straight position during the period of this measurement. Secondly, for bending assessment the sensor output voltage ratio with respect to the reference voltage was measured at different bending angles. The sensor on the test rig was bent at several incremental bending angles degree in 2.5° steps starting from a straight position (assumed to be zero bending) until the fibre was bent at a maximally required angle of 20°. Lastly for repeatability assessment, the fibre was bent several times from 0° to 10° and then back to 0° to study the repeatability of the sensor to give the same output voltage ratio after multiple bending movements of the sensor. The fibre was maintained at each angular position for one minute as the measurement was recorded. The input voltage was also fixed at 3.4 V at all times for both bending and repeatability assessments.

The justifications of the parameters selection of the sensor configuration are presented in the following paragraphs. The right selection of reflector material is important to ensure a good light reflectivity from the source fibres into the reference fibre. A discussion on the fibre separation gap between the source and the sensor fibres is important to explain the needs for dual source fibres for the sensor input; e.g., a gap of up to 4 mm between the source and sensor fibres that was necessary for optimum light reflection into the reference fibre as well as for smooth fibre bending movement would lead to a poor signal reception at the sensor fibre. Lastly, it is also necessary to find a suitable gap so that a maximum light intensity is reflected back from the source fibres into the reference fibre.

### 4.1. Reflector Material Type

**Figure 3.**
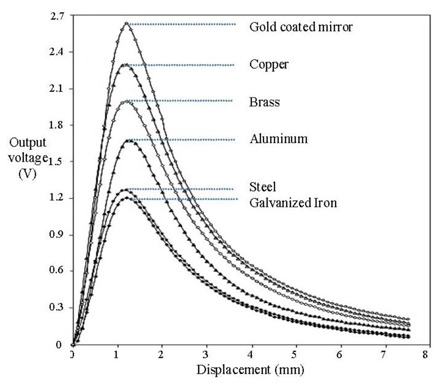
Response of fibre optic reflectivity of different reflector materials [31].

Several types of reflector surface materials can be applied as the reflector element to provide the light reflection for reference fibre signal besides the use of mirror. Optical reflectivity properties for different type of materials have been studied using a red LED light source with 660 nm wavelength. The reflectivity response of gold coated mirror, copper, brass, aluminum, steel and iron obtained from previous investigation [31] is summarized in Figure 3.

It is important to note that the reflectivity of the gold coating surface is dependent on the wavelength of the applied light source. For a visible light source with a wavelength of 600 nm and above, the reflectivity can reach values of up to 90% [32]. On the other hand, for the type of light source in this investigation (green LED: 530 nm), the reflectivity is significantly lower, thus the use of a gold coated mirror is avoided in this application. Steel and iron are more common in the market but their poor reflectivity properties became the main problem. Copper, brass and aluminum are available at relatively low price and it have acceptable reflectivity characteristics, thus they were considered as the reflectors for this sensor configuration.

### 4.2. Fibre Separation Gap between Source Fibres and Sensor Fibre Effect

The transmitted light source power ratio of the sensor fibre due to fibre gap separation between the source fibre and the sensor fibre is quoted based on the connector loss calculation between two fibres. Two different equations were used to estimate the percentage of power loss of the light source as the gap between the source fibre and sensor fibre was increased. Assuming power received at the sensor fibre, *P_r_* and power transmitted from the source fibre, *P_t_*, the first equation is presented as follows [33]:

(1) $$\frac{P_{r}}{P_{t}}=1-\frac{2\times z\times NA}{3\times n_{o}\times d}$$

where *z* is the fibre gap between the source and sensor fibres, *NA* is the numerical aperture of the fibre, *d* is the fibre core diameter and *n_o_* is the refractive index of the medium in between them.

Another approximation is also based on separation loss of two fibre end tips on a joint presented by Tsuchiya as follows [34]:

(2) $$\frac{P_{r}}{P_{t}}=\frac{16K^{2}}{{\left(1+K\right)}^{4}}\cdot \left(1-\frac{z}{4a}\cdot K\cdot {\left(2\cdot \Delta \right)}^{0.5}\right)$$

where the parameter *K* is dependent on the ratio between the fibre core and the medium refractive index, *n_c_*/*n_o_*, *z* is the fibre separation gap, *a* is the core radius and Δ is the refractive index difference between core and cladding (for PMMA fibre, Δ = 1.492 − 1.402 = 0.09). The estimations of the power ratio at different fibre separation gaps between the source and sensor fibres utilizing Equations (1) and (2) are summarized in Table 1.

With the use of Equation (2), the estimated power transmission for a gap of 3 mm between the source fibre and the sensor fibre is less than 3%. The comparison between the theoretical and the experimental values with respect to this longitudinal separation gap has been discussed in our previous study [35]. From the estimation in Table 1, the use of a single source fibre for the sensor configuration presented in this paper will result in a significantly lower light intensity being accepted at the sensor fibre. To ensure a higher light level reception at the sensor fibre, two source fibres were applied in the optical fibre sensor. This was significant because besides the loss due to the fibre gap, the sensor fibre will also experience additional light loss from the bending measurement later.

### 4.3. Distance between the Source Fibres End Tips and Reflector

The optimum distance between the fibre end tips and the reflector applied in this investigation is aimed to achieve a maximum optical power received at the reference fibre. The power ratio of the receiving fibre with respect to the transmitted power for different separation gaps between the fibre end tip and the reflector can theoretically be presented in an equation [36]:

(3) $$\frac{P_{r}(x)}{P_{t}}=\frac{2}{\zeta^{2}}e^{-8/\zeta^{2}}$$

where ζ = 1+2*x*/*z_a_*. In this equation, *z_a_* is the cone vertex distance from the fibre end tips. It was shown in [36] that the collected power at receiving fibre achieved its maximum value at $\zeta =\sqrt{8}$ (e.g., *x*/*z_a_* = 0.9142). For a PMMA fibre type applied in this investigation, the maximum received power was reached at a fibre and reflector separation gap of 1.6 mm (e.g., z_a_ ≅ 1.75 mm as shown by a reflective sensor configuration for vibration application [37] using plastic optical fibre of the same type as applied here. As the percent ratio of the hole area (0.75 mm^2^) by the overall reflector area (9.6 mm^2^) was 7.7%, which is very small, and the hole was in a fixed position on the reflector surface at all time, Equation (3) is presumed to be valid to provide the optimum gap estimation for this sensor.

From the brief discussion provided in Sections 4.1 through 4.3, aluminum foil has been selected as the reflective material considering its reflectivity response and the availability of that material with an adhesive side. The fibre separation gap between the source fibres and the sensor fibre was kept as small as possible. However, as a certain gap was necessary for the optimum light reflection into the reference fibre as well as for a smooth bending movement between the source fibre and sensor fibre holders the fibres was separated a distance between 3 to 4 mm. Finally, the distance between the source fibres and the reflector was set at 1.6 mm simply by using the result from Equation (3).

## 5. Results and Discussion

The experimental results for the bending sensor configuration in this investigation were achieved from a photodetector amplifier and filtering circuit using Labview platform via a multifunction USB-6008 DAQ module (National Instruments, Texas, TX, USA). The results include the power fluctuation test, bending assessment, repeatability assessment and output voltage drift test. From these results, important sensor properties such as sensor sensitivity at different bending angles, sensor working range, acceptable input voltage variation and output linearity were established.

**Figure 4.**
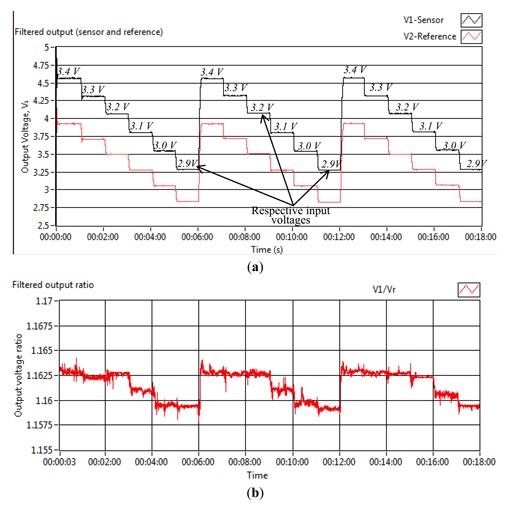
Power fluctuation test—(**a**) actual output voltage; (**b**) output voltage ratio.

Figure 4a,b shows the actual output voltage and output voltage ratio for power fluctuation investigation. Initially, the input voltage was maintained at 3.4 V for 1 min before a decrease of 0.1 V was applied and continued at each additional minute. The input voltage was reduced until 2.9 V. In order to have sufficient light transmission from the source fibres into the reference and sensor fibres, the input voltage was not reduced further. From Figure 4b, it is demonstrated that the output voltage ratio was able to regulate at 0.55% voltage ratio from the initial reading for an input voltage range between 2.9 V and 3.4 V.

**Figure 5.**
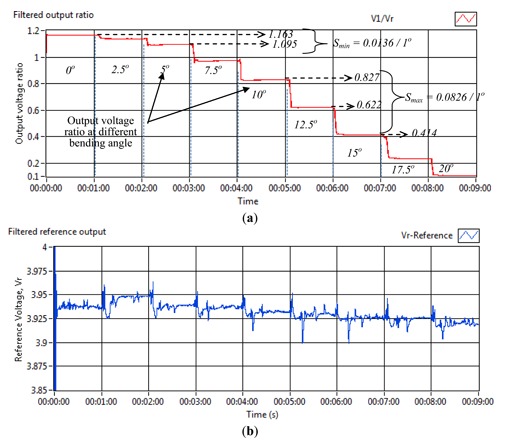
Bending assessment—(**a**) output voltage ratio; (**b**) reference voltage.

The bending test result of the sensor is presented in Figure 5a. The sensor was placed in a straight position at the beginning of the test for 1 min period. Later the bending angle was extended for an additional 2.5° for the next 1 min and so on until 20° of bending was achieved. The actual output voltages of the sensor and reference fibres were recorded to monitor the attenuation of the light intensity at each further 2.5° bending angle.

It is shown in Figure 5a that the output voltage ratio drops more significantly for each additional 2.5° bending angle between 10° and 15° (sensitivity, *S*_max_ = 0.0826/1°) than a bending angle of less than 10°. The output ratio was minimally reduced at a bending of less than 5° (*S*_min_ = 0.0136/1°) which was caused by a wider far field property of the sensor fibre using dual source input [33]. Besides that, as shown in Figure 5b the reference voltage output was also least affected from the fibre bending manipulation, suggesting a reliable compensation signal provided by this sensor configuration.

**Figure 6.**
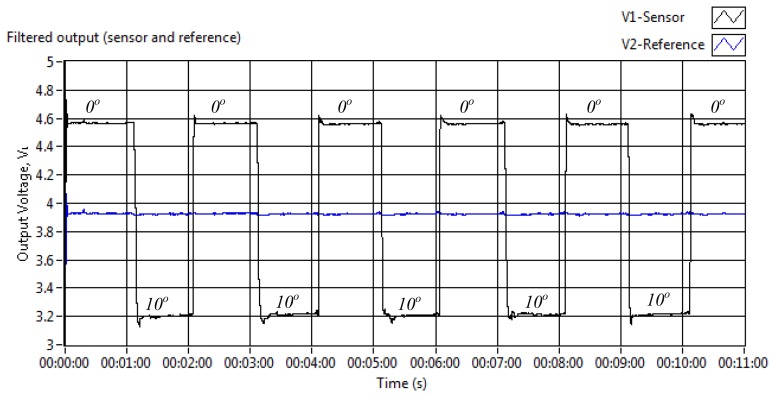
Repeatability test—actual output voltage.

The repeatability test was conducted to study the ability of the rubber tube to hold the fibre holders together during the bending and to maintain the same level of sensor output voltage for each particular bending angle. As shown in Figure 6, the voltage was dropped from 4.57 V to 3.20 V as the sensor was bent from 0° to 10° and the same level of output voltage (thus similarly for output ratio) was reproduced after at least five bending repeatations.

**Figure 7.**
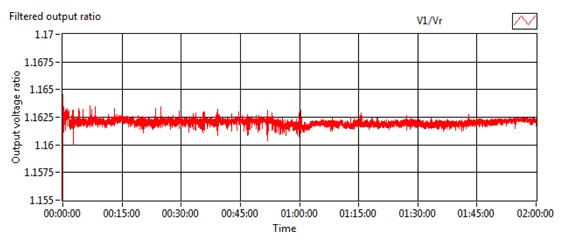
Voltage drift test—output voltage ratio.

The voltage drift test is presented in Figure 7. The sensor output voltage ratio was monitored for a 2 h period to investigate the output voltage ratio drift effect. The drift was found to be at 0.25% from the initial value after being slightly dropped from 1.164 to 1.161 during the 2 h test. This small drift is significant for the optical fibre bending sensor based on intensity modulation as presented in this investigation because a larger drift could potentially lead to miscalculation of the bending angle, especially for a long period of spine bending monitoring.

**Figure 8.**
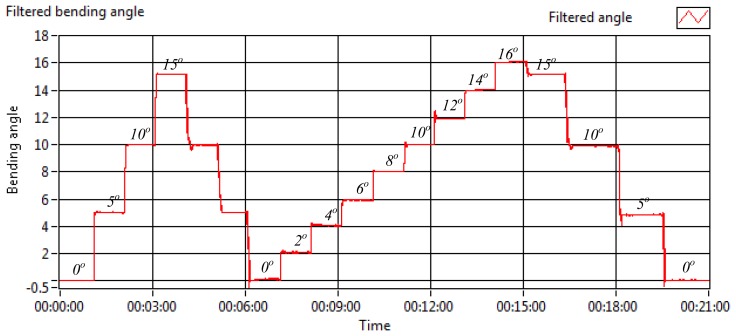
Bending measurement—bending angle (°).

To show the ability of the sensor to provide a bending measurement for the intended application, the measurement was made by determining the output ratio at several pre-determined angles before a linear relationship between the output ratio and the bending angle was made. This relationship was applied only for this preliminary investigation as an actual relationship between the output ratio and the bending angle will be applied using fibre tilt angle loss estimation as discussed in [35]. In this measurement, the sensor was bent starting from a straight position (0°) to 5°, 10° and later to 15° before it was continued for decreasing angles of the same bending angle position as shown in Figure 8. As this scale was too large for the spine application, the measurement was carried on for a smaller bending scale of 2° until 16° of bending. The bending reading between 0° and 6° was slightly less consistent than the larger bending which was explicable due to the poor sensitivity of the sensor at a smaller bending range.

From the assessment results in Figures 4, 5, 6, 7 and 8, some important properties of the sensor under investigation are summarized in Table 2. The effect of the other disturbances such as temperature variation and external light coupling were minimized in the current investigation results by conducting the measurement in an ambient room temperature (between 18 °C to 23 °C) and minimum surrounding light during the measurement period. The room temperature variation will affect the light intensity at some point but this effect is presumed to be minimized with the compensation technique described here. The reason is both reference and sensor outputs were supplied by the same light sources before these values were normalized for the bending measurement. Besides that, it was also presumed that the body temperature does not directly influence the light intensity because in this case only the fibre cable has a direct contact with the human body while the light source was placed in a distant location.

As a general guideline for the minimum requirement of the sensor resolution and its full scale deflection specifically for the application of the spine bending and movement monitoring, it is significant to briefly review the typical range of motion of the human spine based on previous studies on human spine motion conducted by recognized physiotherapists. The summary of the range of motion of the segmental of the human spine is presented in Table 3.

As shown in Table 3, the bending angles for the human spine, particularly the lumbar spine, for lateral flexion, flexion and extension movements range between 1° and 13°. The use of a single point optical fibre bending sensor presented in this study has a working range of up to 20° and it is capable of measuring the bending angle at a minimum resolution of 2° at each measurement point. However, for an overall assessment of the spine bending angle at each level of the lumbar spine (or up to cervical spine levels), a distributed optical fibre sensor can be implemented using the same compensation approach presented here.

From the spine bending application perspective using the proposed sensor, it was noticed that the sensor dimension was about 7 mm in diameter and 40 mm in length (end to end of fibre holders). At this point, the bending angle in flexion direction between 0° and 20° can be measured continuously at one particular point. The main objective of this article is to highlight the potential of the proposed sensor configuration to compensate for the input power fluctutation at a minimum output drift and a good repeatability. Further improvements are in progress to minimize the sensor dimensions, especially its length, to permit additional sensor point installation for multiple location bending assessment along the human spine.

## 6. Conclusions

The implementation of an optical fibre bending sensor using an intensity modulation approach with power fluctuation compensation has been presented. The compensation was obtained from the output voltage ratio between the sensor output and the reference signal. The reference signal was collected at the end tip of the reference fibre due to light reflection via an aluminum foil surface fixed at a few gaps away from the source fibres end tips. Using the compensation technique described above, a resolution of less than 2° can be achieved, with an output drift of 0.25% during 2 h measurement, 0.55% of power fluctuation changes for an input voltage variation between 2.9 V and 3.4 V as well as sensor working range between 0° and 20°. This sensor configuration meets the required range of motion of the human spine as defined by Bogduk [38] for continuous monitoring of the spine bending at one measurement point.

## Figures and Tables

**Table 1.** Transmitted power ratio estimation due to fibre separation gap.

**Fibre Separation,*z* (mm)**	**Power Ratio**, *P_r_/P_t_***(%)**	

	$\left(1-\frac{2\times z\times NA}{3\times n_{o}\times d}\right)$	$\frac{16K^{2}}{{\left(1+K\right)}^{4}}\cdot \left(1-\frac{z}{4a}\cdot K\cdot {\left(2\cdot \Delta \right)}^{0.5}\right)$
0.25	91.50	84.90
*0.5*	83.00	77.44
*0.75*	74.50	70.00
*1.0*	66.00	62.57
*1.25*	57.48	55.07
*1.5*	48.98	47.61
*1.75*	40.48	40.15
*2.0*	31.97	32.70
*2.5*	14.97	17.79
*3.0*	*<1.00*	2.87

**Table 2.** Optical fibre bending sensor properties of this investigation.

**Sensor Properties**	**Measured Value**
Power fluctuation (input voltage of 2.9 V–3.4 V)	0.55%
Sensitivity (between 0° and 5°)	*S*_min_ = 0.0136/1°
Sensitivity (between 10°and 15°)	*S*_max_ = 0.0826/1°
Resolution	<2°
Working range (flexion only)	0°–20°
Output drift	0.25%/2 h

**Table 3.** Ranges of segmental motion for different motion types [38].

**Level of Lumbar Spine**	**Mean Range (°)**		

	**Lateral Flexion**	**Flexion**	**Extension**
L1-2	5	8	5
L2-3	5	10	3
L3-4	5	12	1
L4-5	3	13	2
L5-S1	2	9	5

## References

[1] Snell R.S. (2004). The Back. Clinical Anatomy by Regions.

[2] Croft P.R., Macfarlane G.J., Papageorgiou A.C., Elaine T., Silman A.J. (1998). Outcome of low back pain in general practice: A prospective study. Br. Med. J..

[3] Spenkelink C.D., Hutten M.M.R., Hermens H.J., Greitemann B.O.L. (2002). Assessment of activities of daily living with an ambulatory monitoring system: A comparative study in patients with chronic low back pain and non-symptomatic controls. Clin. Rehabil..

[4] Link C.S., Nicholson G.G., Shaddeau S.A., Robert B., Gossman M.R. (1990). Lumbar curvature in standing and sitting in two types of chairs: Relationship of hamstring and hip flexor muscle length. Phys. Ther..

[5] Mellin G.P. (1986). Accuracy of measuring lateral flexion of the spine with a tape. Clin. Biomech..

[6] Burdett R.G., Brown K.E., Fall M.P. (1986). Reliability and validity of four instruments for measuring lumbar spine and pelvic positions. Phys. Ther..

[7] Mannion A.F., Knecht K., Balaban G., Dvorak J., Grob D. (2004). A new skin-surface device for measuring the curvature and global and segmental ranges of motion of the spine: Reliability of measurements and comparison with data reviewed from the literature. Eur. Spine J..

[8] Whittle M.W., Levine D. (1999). Three-dimensional relationships between the movements of the pelvis and lumbar spine during normal gait. Hum. Mov. Sci..

[9] Crawford R.J., Price R.I., Singer K.P. (2009). The effect of interspinous implant surgery on back surface shape and radiographic lumbar curvature. Clin. Biomech..

[10] Ahmadi A., Maroufi N., Behtash H., Zekavat H., Parnianpour M. (2009). Kinematic analysis of dynamic lumbar motion in patients with lumbar segmental instability using digital videofluoroscopy. Eur. Spine J..

[11] Shope T.B. (1996). Radiation-induced skin injuries from fluoroscopy. Radiographics.

[12] Djordjevich A., He Y. (1999). Thin structure deflection measurement. IEEE Trans. Instrum. Meas..

[13] Donatell G.J., Meister D.W., O'Brien J.R., Thurlow J.S., Webster J.G., Salvi F.J. (2005). A simple device to monitor flexion and lateral bending of the lumbar spine. IEEE Trans. Neural Syst. Rehabil. Eng..

[14] Janssen W.G.M., Bussmann J.B.J., Horemans H.L.D., Stam H.J. (2005). Analysis and decomposition of accelerometric signals of trunk and thigh obtained during the sit-to-stand movement. Med. Biol. Eng. Comput..

[15] Rowe P.J., White M. (1996). Three dimensional lumbar spinal kinematics during gait, following mild musculo-skeletal low back pain in nurses. Gait Posture.

[16] Williams J.M., Haq I., Lee R.Y. (2010). Dynamic measurement of lumbar curvature using fibre-optic sensors. Med. Eng. Phys..

[17] The Validation of Measurend Shape Tape for Measuring Joint Angles. http://www-archive.ece.queensu.ca/directory/faculty/Morin/publications/CMBEC_02_shapetape.pdf.

[18] Vogt L., Banzer W. (1999). Measurement of lumbar spine kinematics in incline treadmill walking. Gait Posture.

[19] Dunne L.E., Walsh P., Hermann S., Smyth B., Caulfield B. (2008). Wearable monitoring of seated spinal posture. IEEE Trans. Biomed. Circuit. Syst..

[20] Kuang K.S.C., Cantwell W.J., Scully P.J. (2002). An evaluation of a novel plastic optical fibre sensor for axial strain and bend measurements. Meas. Sci. Technol..

[21] Wei H.L., Li W.C., Wen F.X., Yung C.C. (2012). A sensing element based on a bent and elongated grooved polymer optical fiber. Sensors.

[22] Yuan L., Pan J., Yang T., Han G. (1993). Analysis of the compensation mechanism of a fiber-optic displacement sensor. Sens. Actuator. A.

[23] Yuan L. (1998). Automatic-compensated two-dimensional fiber-optic sensor. Opt. Fiber Technol..

[24] Ko W.H., Chang K.M., Hwang G.J. (1995). A fiber-optic reflective displacement micrometer. Sens. Actuators A.

[25] Yuan L., Anping Q. (1991). Fiber-optic diaphragm pressure sensor with automatic intensity compensation. Sens. Actuators A.

[26] Vallan A., Casalicchio M.L., Olivero M., Perrone G. (2012). Assessment of a dual-wavelength compensation technique for displacement sensors using plastic optical fibers. IEEE Trans. Instrum. Meas..

[27] Thorlabs: Pellicle Beamsplitters. http://www.thorlabs.de/newgrouppage9.cfm?objectgroup_id=898.

[28] Thorlabs: Polka Dot Beamsplitters. http://www.thorlabs.de/newgrouppage9./cfm?Objectgroup_id=1110.

[29] Thorlabs: Visible Beamsplitters. http://www.thorlabs.de/newgrouppage9.cfm?objectgroup_id=914.

[30] Ziemann O., Krauser J., Zamzow P.E., Daum W. (2008). Passive Components for Optical Fibers: POF Couplers. POF Handbook: Optical Short Range Transmission Systems.

[31] Binu S., Mahadevan Pillai V.P., Chandrasekaran N. (2007). Fibre optic target reflectivity sensor. Opt. Quantum Electron..

[32] Inradoptics: Spectral Reflectivity Curves. http://www.inradoptics.com/optics-resources/optics-design/spectral-reflectivity-curves.

[33] Ziemann O., Krauser J., Zamzow P.E., Daum W. (2008). Passive Components for Optical Fibers: Basis for Calculating Connector Losses. POF Handbook: Optical Short Range Transmission Systems.

[34] Tsuchiya H., Nakagome H., Shimizu N., Ohara S. (1977). Double eccentric connectors for optical fibers. Appl. Opt..

[35] Zawawi M.A., O'Keeffe S., Lewis E. (2013). Plastic optical fibre sensor for spine bending monitoring. J. Phys..

[36] Faria J.B. (1998). A theoretical analysis of the bifurcated fiber bundle displacement sensor. IEEE Trans. Instrum. Meas..

[37] Perrone G., Vallan A. (2009). A low-cost optical sensor for noncontact vibration measurements. IEEE Trans. Instrum. Meas..

[38] Bogduk N. (1997). Movement of the Lumbar Spine. Clinical Anatomy of the Lumbar Spine and Sacrum.

